# Coping with healthcare costs for chronic illness in low-income and middle-income countries: a systematic literature review

**DOI:** 10.1136/bmjgh-2019-001475

**Published:** 2019-08-21

**Authors:** Adrianna Murphy, Catherine McGowan, Martin McKee, Marc Suhrcke, Kara Hanson

**Affiliations:** 1 Centre for Global Chronic Conditions, Department of Health Services Research and Policy, Faculty of Public Health and Policy, London School of Hygiene and Tropical Medicine, London, UK; 2 Department of Public Health, Environments and Society, Faculty of Public Health and Policy, London School of Hygiene & Tropical Medicine, London, UK; 3 Humanitarian Public Health Technical Unit, Save the Children UK, London, United Kingdom; 4 Centre for Health Economics, University of York, York, UK; 5 Luxembourg Institute of Socio-economic Research (LISER), Belval, Luxembourg; 6 Department of Global Health Development, Faculty of Public Health and Policy, London School of Hygiene and Tropical Medicine, London, UK

**Keywords:** health systems, health economics, health insurance, public health, systematic review

## Abstract

**Background:**

Experiencing illness in low-income and middle-income countries (LMICs) can incur very high out-of-pocket (OOP) payments for healthcare and, while the existing literature typically focuses on levels of expenditure, it rarely examines what happens when households do not have the necessary money. Some will adopt one or more ‘coping strategies’, such as borrowing money, perhaps at exorbitant interest rates, or selling assets, some necessary for their future income, with detrimental long-term effects. This is particularly relevant for chronic illnesses that require consistent, long-term OOP payments. We systematically review the literature on strategies for financing OOP costs of chronic illnesses in LMICs, their correlates and their impacts on households.

**Methods:**

We searched MEDLINE, EconLit, EMBASE, Global Health and Scopus on 22 October 2018 for literature published on or after 1 January 2000. We included qualitative or quantitative studies describing at least one coping strategy for chronic illness OOP payments in a LMIC context. Our narrative review follows Preferred Reporting Items for Systematic Reviews and Meta-Analyses reporting guidelines.

**Results:**

Forty-seven papers were included. Studies identified coping strategies for chronic illness costs that are not traditionally addressed in financial risk protection research (eg, taking children out of school, sending them to work, reducing expenditure on food or education, quitting work to give care). Twenty studies reported socioeconomic or other correlates of coping strategies, with poorer households and those with more advanced disease more vulnerable to detrimental strategies. Only six studies (three cross-sectional and three qualitative) included evidence of impacts of coping strategies on households, including increased labour to repay debts and discontinuing treatment.

**Conclusions:**

Monitoring of financial risk protection provides an incomplete picture if it fails to capture the effect of coping strategies. This will require qualitative and longitudinal research to understand the long-term effects, especially those associated with chronic illness in LMICs.

**Key questions**

**What is already known?**

- Extensive research shows that chronic illnesses can increase household expenditure, but the range of strategies households use to cope with chronic out-of-pocket healthcare payments, the determinants of these strategies and their long-term impact on households, are less well understood.

**What are the new findings?**

- Households with chronic illnesses employ a range of strategies not traditionally captured in financial protection research, for example, taking children out of school or stopping treatment, and there is also heterogeneity within single types of coping strategies (eg, sale of productive vs non-productive assets).
- Very few studies have considered determinants of detrimental coping strategies, while qualitative work suggests coping strategies can lead to long-term indebtedness, exacerbated illness, stigma and strained social relationships.

**What do the new findings imply?**

- Efforts to monitor financial protection must consider a wider range of strategies used by households with chronic illness to cope with healthcare costs, as a failure to account for their impacts will underestimate the gap in financial risk protection.
- More qualitative and longitudinal research is needed to inform ways to support vulnerable groups and more comprehensively monitor financial risk protection, particularly in LMICs underrepresented in health systems research.

## Background

Financial risk protection has long been recognised as a core objective of universal health coverage (UHC), now included explicitly in the United Nations Sustainable Development Goal (SDG) 3 on health and well-being. Thus, the relevant target is to ‘Achieve universal health coverage, including financial risk protection, access to quality essential healthcare services….for all’.^1^ Yet, accurately measuring financial risk protection is a challenge. The 2017 WHO and World Bank (WB) Global Monitoring Report on UHC (entitled ‘Tracking Universal Health Coverage’) proposes measures such as ‘catastrophic’ health expenditures (expenditures that are higher than a given proportion of household resources) and ‘impoverishing’ health expenditures (expenditures that push households below the poverty line), with the goal of protecting 100% of the population from both of these.^2^ However these measures may underestimate the full economic impact of healthcare expenditure on many households as they fail to take into account the strategies employed to cope with these costs.^3 4^ Few people in many low-income and middle-income countries (LMICs) are able to pay large sums from current income or to draw on savings^5 6^ to pay healthcare bills, and so must resort to alternative strategies, including borrowing money or selling assets.^4 6^ Such responses are variously termed ‘coping strategies’ or ‘distress’^7–9^ or ‘hardship financing’.^6^ Although they allow households to pay for care for short periods, they conceal potential longer-term economic consequences^4^ (eg, those arising from repayment of high interest loans, or lost returns after sale of productive assets), which are not captured by conventional measures of financial protection. These longer-term consequences, both economic and non-economic, may be severe, including inability to invest in education,^10^ reducing food consumption, compromising on timely medical care or returning to work before having fully recovered^11^ in order to meet financial obligations. All of these risks push households further into poverty and trap them there.^4 12^ Flores *et al* estimated that the poverty head count in India would increase by 0.6 percentage points (a large absolute increase given India’s population) if the use of savings, borrowing money or selling assets to finance healthcare was accounted for.^4^ However, the range of strategies employed by households likely extends beyond those commonly cited, with each strategy having a different impact.

The consideration of financial risk protection is particularly relevant given increased recognition of the economic burden of chronic illnesses, in particular non-communicable diseases (NCDs, eg, cardiovascular disease (CVD)),^7^ and chronic infectious diseases (eg, HIV^13^) on patient households. The advent of modern, life-sustaining treatment means that affected households must make regular, often lifelong, out-of-pocket (OOP) payments for follow-up care and drug treatment. This contrasts with the one-off or time-limited payments needed for acute conditions. The challenges are exacerbated by how chronic illnesses often diminish ability to work, with consequent loss of income.^14 15^ While there is now extensive research on how chronic illnesses can increase household expenditure, the range of strategies households use to cope with chronic OOP healthcare payments, and their long-term impact, are less well understood. Given the increasing prevalence of chronic NCDs in LMICs, a failure to account for these strategies and their impacts will underestimate the gap in financial risk protection,^4^ and prevent accurate evaluation of the effects of policy responses designed to improve financial risk protection.^16^

We set out to review systematically the literature on strategies used by households to cope with healthcare costs for chronic illnesses. Our objective is to review current knowledge and identify evidence gaps regarding the range of strategies employed for financing healthcare costs of chronic illnesses, their determinants, and the potential long-term social, financial and health impacts.

## Methods

This review has been registered on the PROSPERO international prospective register of systematic reviews, with the record number CRD42018113014. We searched key health sciences, health economics and public health databases—MEDLINE, EconLit, EMBASE and Global Health, and one multidisciplinary database, Scopus, on 22 October 2018. A sample search strategy (from MEDLINE search) is included in box 1. A table outlining our inclusion and exclusion criteria is included as online supplementary file 1. We searched for literature published in English on or after 1 January 2000. Initial screening on title and abstract was carried out by AM and CM and sought to identify potentially relevant qualitative or quantitative studies describing at least one coping strategy for dealing with chronic illness in a LMIC context. (Countries were included if they were defined as LMIC by WB country classifications^17^ at any point from 1 January 2000 to the day of the search). Papers from high-income countries or those focusing exclusively on general healthcare or acute illness were excluded. Papers that specified a focus on both acute and chronic illnesses were included; however, only those data reporting on coping with chronic illnesses were extracted. Papers that did not specify the type of illness (eg, general healthcare) were excluded. Disagreements about inclusion of sources were discussed by AM and CM and were included after consensus was reached. Data from full-text sources were extracted by AM using the following headings: first author, year, country, disease focus, study type, any coping strategies identified, factors affecting coping strategy choice, socio-economic status (SES) differences in coping strategy choice and impacts of coping strategy. We anticipated that studies would not be sufficiently homogenous to either justify or permit carrying out a meta-analysis. We used a narrative synthesis approach to review evidence from included studies and the implications of different study approaches for findings. Our review report follows the Preferred Reporting Items for Systematic Reviews and Meta-Analyses (PRISMA).^18^

10.1136/bmjgh-2019-001475.supp1 Supplementary data

Box 1 **Sample search strategy (Medline)**

**MEDLINE**

- exp Fees, Medical/
- exp Hospital Costs/
- (“health care” or “healthcare” or “medical cost*” or “medical fee*” or “hospital cost*” or “hospital fee*” or “health expenditure*” or “catastrophic adj2 expenditure*” or “catastrophic adj2 spending” or “out-of-pocket” or “health shock*”).ab. or (“health care” or “healthcare” or “medical cost*” or “medical fee*” or “hospital cost*” or “hospital fee*” or “health expenditure*” or “catastrophic adj2 expenditure*” or “catastrophic adj2 spending” or “out-of-pocket” or “health shock*”).ti.
- (coping or cope or “distress finance*” or “hardship finance*” or “consumption smoothing” or “income smoothing” or “consumption insurance”).ab. or (coping or cope or “distress finance*” or “hardship finance*” or “consumption smoothing” or “income smoothing” or “consumption insurance”).ti.
- (household* or family or families).ab. or (household* or family or families).ti.
- 1 or 2 or 3
- 4 and 5 and 6

### Patient and public involvement

Patients were not involved in the design or conduct of this systematic review.

## Results

### Study context and methods

**Figure 1:**
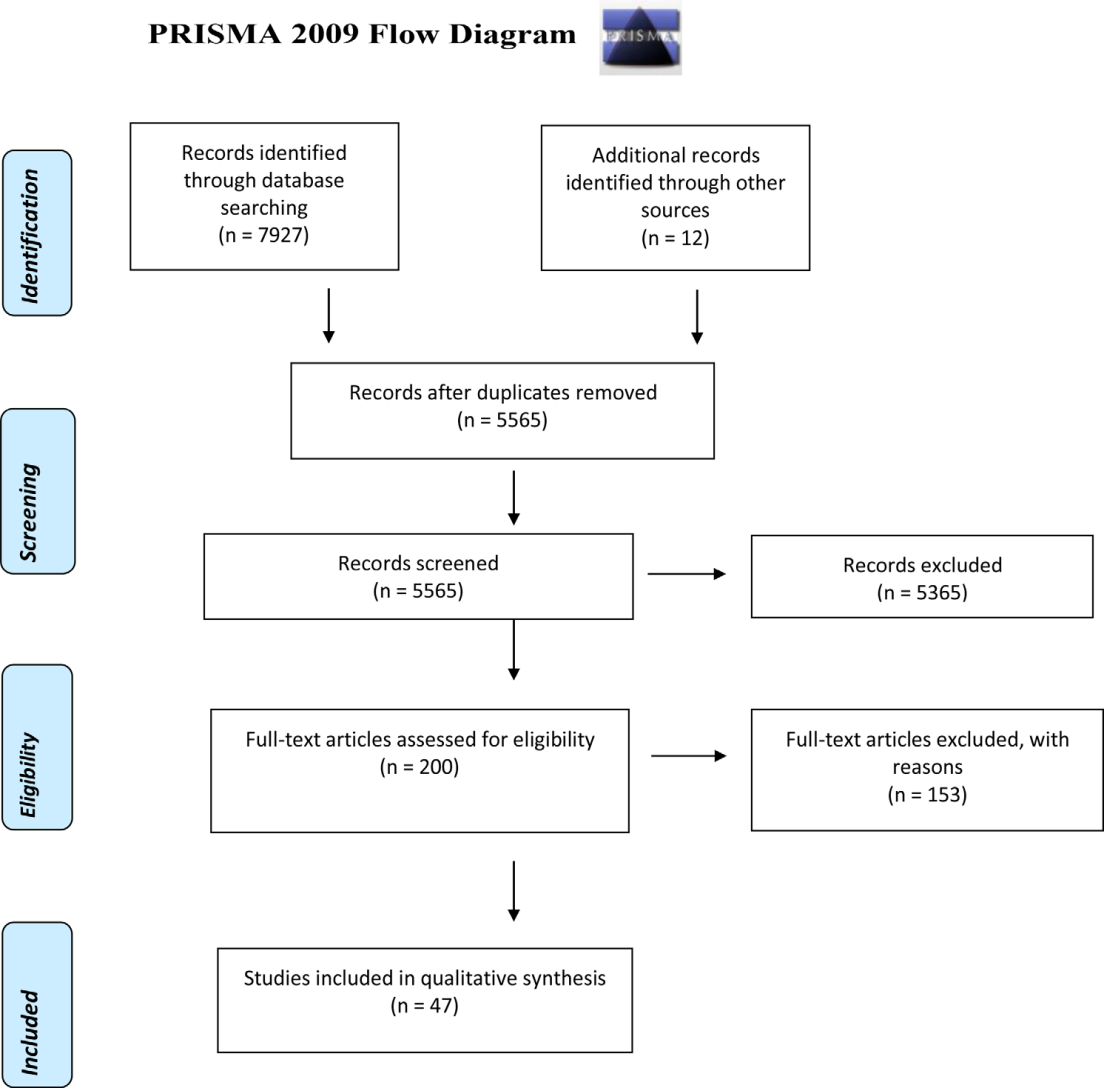
The Preferred Reporting Items for Systematic Reviews and Meta-Analyses (PRISMA) flow chart.

The PRISMA flow chart is included in figure 1. Forty-seven papers met our inclusion criteria and were included in the narrative synthesis (table 1 and online supplementary file 2).

10.1136/bmjgh-2019-001475.supp2 Supplementary data

**Table 1:** Number of included studies by geographical region, disease category and type of study

	Number of studies*	Per cent of all studies included (%)
World Bank region		
Sub-Saharan Africa	20	43
East Asia and Pacific	11	23
South Asia	12	26
Europe and Central Asia	4	9
Latin America and Caribbean	3	6
Middle-East and North Africa	1	2
Disease category		
HIV/AIDS or TB	26	55
CVD and hypertension	8	17
Diabetes	3	6
Cancer	3	6
Physical disability	2	4
Mental disorder	1	2
Renal disease	1	2
Chronic obstructive pulmonary disease	1	2
Sickle cell disease	1	2
Combined chronic diseases	4	9
Study type		
Cross-sectional	32	68
Longitudinal	4	9
Qualitative	8	17
Mixed-methods	3	6

*Numbers for region and disease category add to more than 47 as some studies covered more than one region or disease category.

CVD, cardiovascular disease; TB, Tuberculosis.

Characteristics of the included studies, including WB region, disease type and type of study are summarised in table 1. Almost all included studies relied on cross-sectional surveys (n=32). The remainder used either prospective longitudinal studies (n=4), qualitative methods (n=8) or mixed methods (n=3).

### Coping strategies identified

While most studies used the broad categories of strategies for coping with healthcare costs commonly covered in international household surveys (eg, using income/savings, borrowing money or selling assets) some drilled down to distinguish between types of borrowing and asset sales.^19–28^ For example, some studies examined who households borrowed from, noting that those forced to borrow from moneylenders faced higher interest rates than those borrowing from family or friends.^25 26 28^ The diversity of strategies was revealed in a study of households containing women with breast cancer. Although almost 85% and 75%, respectively, were able to borrow at low interest rates or obtain support from families and friends, most also had to pawn personal items such as jewellery, borrow money at high interest rates or sell financial assets (shares, gold, etc) or economically productive assets, such as cattle. Most used a combination of strategies.^25^ A study of households with non-specific chronic conditions, also in India, found evidence that households may mortgage assets to pay for healthcare costs.^29^

Several studies provided evidence of other strategies to cope with healthcare costs that may have detrimental long-term impacts. These included: taking children out of school or sending them to work,^13 21 25 27 30–32^ reducing expenditure on food, education or social activities,^13 28 32–37^ institutionalising a child or adult patient,^13^ taking on extra work,^13 32 38 39^ quitting work to take on the role of caregiver,^13 40^ replacing labour (eg, a wife taking on ill husband’s work),^41^ taking a donation from a healthcare provider,^41^ or moving to cheaper or free accommodation.^21^ Coping strategies with more immediate potential impacts on health were also identified.^21 28 34 42^ For example, patients with HIV/AIDS in Zimbabwe reported coping with the costs of their treatment by: seeking alternative treatment, seeking cheaper treatment, delaying treatment or stopping treatment altogether.^34^ Thus, such responses may, in the long term, be detrimental by increasing the risk of future costs, creating a downward spiral.

### Determinants of coping strategies

Only 20 of the 47 included provided some evidence of the factors correlated with different coping strategies. These included demographic factors, with one study of households with chronic illness in Russia, which found that female-headed households and households with a higher number of old people were more likely to receive gifts or family transfers to finance healthcare (although the study does not clarify a threshold for ‘old’).^43^ Patient exit interviews in South Africa found that patients with HIV or TB were more likely to borrow money or sell assets to pay for healthcare than were obstetric patients, and that borrowing money was more common among patients from rural versus urban areas.^44^ More severe disease was associated with an increased likelihood of distress financing generally among patients with cancer,^25^ and of cattle sale among patients with HIV.^45^ Those with CVD^46^ or angina^7^ were more likely to borrow or sell assets to pay for healthcare than those without these diseases. The extent of a household's social capital was also found to influence coping strategy (eg, receiving support from a local Non-governmental organisation (NGO) as opposed to having to sell assets or compromise treatment quality).^34^

Among those studies that found an association between coping strategy and socioeconomic status, poorer households are more likely to engage in detrimental coping strategies such as taking high-interest loans, sale of assets, withdrawing children from school and reducing food consumption, while wealthier households are more likely to rely on insurance, income or savings.^8 25 28 29 47^ (One study found that middle-class households were more likely to sell assets than poor or rich households.^48^) In Iranian households containing someone with cancer, those who had experienced catastrophic spending were more likely to borrow money or sell property.^49^ Perhaps unsurprisingly, patients with HIV/AIDS in Namibia who had private insurance were less likely to take grants or gifts, sell assets, decrease consumption, or take loans to finance healthcare costs.^36^

Households in Punjab, India containing a woman with breast cancer were significantly more likely to report reduced expenditure on food, education and social events, as well as early entry into the labour market if the household was poorer.^25^ On the other hand, one study of households containing patients living with HIV/AIDS in Zimbabwe found that those in poorer households more often relied on social resources, such as support from other households, community groups or local NGOs, perhaps because they had higher social capital.^34^

### Impacts of coping strategies

Only six studies included some evidence of the long-term impacts of coping strategies on patient households. In three different cross-sectional studies, participants reported using multiple sources such as asset sale and increased labour to repay debts from having borrowed money,^50^ discontinuing treatment due to cost^23^ and depleting their savings to pay for healthcare costs.^24^ One qualitative study of patients with acute coronary syndrome included interviews with families about coping strategies at the time of the event and during the subsequent 6-month period. It found that families who borrowed money from moneylenders to finance their healthcare were often exploited, with seizure of property deeds or other assets used as security when families were unable to repay, leading to prolonged, or lifelong, indebtedness. Patients' families also faced being stigmatised as 'charity cases'.^21^ In Sri Lanka, another qualitative study found that many households containing patients with diabetes had to sell income-generating assets, leading to reduced future household earnings. The burden was further exacerbated when households could no longer afford treatment, leading to complications of diabetes and further costs.^27^ Finally, a qualitative study with patients with TB in China found that engaging in strategies such as borrowing, selling assets or receiving transfers or donations resulted in household resources being exhausted, compromises made on medications, strained social relationships, and feelings of stigma and shame.^41^

## Discussion

The United Nations has committed to achieving UHC for all, including financial risk protection, with additional commitments in relation to reducing the burden of NCDs.^51^ Important work has been done to show how measures to expand insurance coverage in LMICs, especially to the poorest families and those with children and older adults, can achieve the greatest improvements in financial protection.^52–54^ However, efforts to monitor progress towards UHC will be incomplete without recognising the range of types of coping strategies employed by households to finance healthcare expenditures, their potential detrimental impacts on household health and economic security, and those groups in the population that are most vulnerable. These include those with chronic illness in many LMICs, where households can face long-term, and often lifelong, OOP payments for healthcare. Our review highlights gaps in research on this topic.

First, most widely used surveys, including the World Health Survey^55^ and WHO Study on Global AGEing and Health,^56^ attempt to estimate the prevalence of a limited set of coping strategies (current income, savings, health insurance, selling items, borrowing from friends or family, borrowing from someone else, and ‘other’). Those surveys, and similar others, have facilitated important multicountry research on the prevalence of detrimental coping strategies.^6 43 57^ However, our review has shown that households with chronic illnesses employ a range of strategies not traditionally captured in those metrics, for example, taking children out of school. The positive impact that childhood education can have on a household’s long-term economic prospects, and the detrimental impact that low education has on the economic development of societies, generally, are well established.^58–60^ Completion of primary and secondary education for all children is another SDG target,^1^ and the WB’s Human Capital programme aims to invest in young people’s education and skill-building as a means to reduce poverty and inequality.^61^ In countries where primary and secondary education are not provided freely, competing demands for household financial resources, such as debt incurred from OOP payments for healthcare, may compromise progress towards these goals.

Coping strategies with a more direct impact on the health of the household member suffering chronic illness were also identified, and these may have adverse longer-term economic repercussions for the households. Stopping or delaying treatment, or seeking alternative treatment for conditions and diseases like hypertension, CVD and diabetes, can increase the risk of acute events such as myocardial infarction^62^ (which are costlier to treat and require hospitalisation), or of death. Future research on financial risk protection for households with chronic illness should include a broader range of potential coping strategies or allow for open-ended questions about coping with healthcare costs.

Second, our review supports the conclusion that there is heterogeneity within single types of coping strategies used for chronic illness costs. Knowing whether, in the case of borrowing, loans were high interest,^63 64^ whether assets sold were productive,^65^ or whether assets were essential to current consumption or surplus to requirements,^9^ are crucial in understanding the potential long-term impact on families of patients. For example, previous research on coping with costs of general healthcare in India showed that borrowing is primarily from informal moneylenders who lend at high rates of interest,^12^ which may drive poorer households into debt bondage.^35 66^ Collecting more detail on commonly reported strategies among households with chronic illness should be a priority for future research.

Third, our review highlights the need for further research on determinants of coping strategies, which can help to focus research and policy initiatives on particularly vulnerable populations. The research we reviewed provides some insights, although some may seem predictable, such as evidence that advanced disease is associated with an increased likelihood of distress financing among patients with cancer in India.^25^ It may also seem predictable that poorer households are more likely to engage in potentially detrimental coping strategies, but contradictory evidence that patients with HIV/AIDS in poorer communities of Zimbabwe were more likely to take advantage of social resources, such as support from other households and local NGOs to finance health expenditures, shows that it is not inevitable. The potential for measures to strengthen these networks, or at least avoid those that undermine them, should be explored as a means to improve protection for households, as well as to ensure that new policies, in any sector, do not undermine them.

Fourth, and perhaps most important for understanding the impact of healthcare costs for chronic illnesses, our review uncovered only minimal evidence of the potential long-term impacts of coping strategies on households. Perhaps the strongest contribution to this evidence was made by qualitative research, providing a richness of data difficult to achieve with quantitative methods, with details of how, for example, the burden of healthcare debt can exacerbate the poor health of those with diabetes, resulting in increased risk for acute disease phases and higher healthcare costs.^27^ It is likely that many of the coping strategies identified such as taking high-interest loans or selling productive assets will have a more severe impact on poorer households, further exacerbating inequity in health and economic well-being. It is also possible that some strategies are more likely to negatively affect women and girls in the household, for example, leaving work to take on caregiving duties or withdrawing from school. More qualitative work is needed to understand these impacts. To complement cross-sectional approaches however, whether quantitative or qualitative, longitudinal research is required to better understand long-term effects of coping strategies, including those on socioeconomic and gender equity. We did not identify any longitudinal studies of the long-term impacts of coping strategies in our review. Future efforts to collect longitudinal data might include research that spans generations, given that several of the coping strategies identified can be expected to have important intergenerational consequences, and the emerging body of research on the health effects of intergenerational mobility.^67^ This could shed light on the complex and dynamic processes that influence how households will be affected by healthcare costs.^68^

Finally, our review uncovered gaps in the diseases and geographical regions included in existing research. The majority of studies included in our review focused on HIV or TB. While these continue to be major contributors to disease burden in many LMICs, other chronic NCDs, including CVD and cancer are increasingly important in these countries. These are not sufficiently addressed in the literature to date.^15^ While the available evidence regarding coping strategies for HIV/TB may be informative for NCDs, it likely does not accurately reflect the burden of coping with costs for chronic NCDs given the absence of external aid funding for chronic NCDs. There is also an increasing prevalence of chronic multimorbidities in LMICs, for example, HIV and CVD, which impose an even greater economic burden.^69^ The almost complete absence, save one study, on coping with the costs of mental disorders highlights the failure to recognise the increasing health and economic burden of mental disorders globally,^70^ and the need for more research in this area. While our review suggests that there is at least some research on strategies for coping with costs of chronic illness in every region containing LMICs, there are many countries that are not represented at all, or where no more than one study has been conducted. More research is required from all LMICs to inform nationally relevant policy responses.

### Limitations

Our review did not explore another limitation of traditional measures of financial risk protection, that is the economic burden faced by households who forego care altogether due to costs. Understanding the extent to which OOP costs prevent households from seeking necessary care is indeed another crucial indicator of UHC, and suggestions for how to incorporate that in monitoring efforts have been made by others.^3^ We also did not explore how households cope with the psychosocial burden of healthcare expenditures, such as effects of costs on the mental health of patients, caregivers and families, which may be exacerbated by coping strategies (for example by stigma associated with borrowing money). Approaches for including this burden in more comprehensive monitoring of financial risk protection should be considered.

We only included English-language papers so we may have missed relevant papers published in other languages, potentially leading to underrepresentation of countries where English is not used.

## Conclusion

Although the potentially detrimental long-term impacts of coping strategies are widely acknowledged,^4 6 9 63^ there is relatively little understanding of different types of coping strategies for chronic illnesses, their determinants and the long-term impacts on households. Our review identifies coping strategies not normally captured by international research on financial protection. It also suggests some potential determinants of coping strategies for chronic illness costs such as socioeconomic status and disease severity, and negative long-term impacts on household health and socioeconomic status. However, more qualitative and longitudinal research, including studies spanning generations, is needed to inform ways to support vulnerable groups and more comprehensively monitor financial risk protection, particularly in LMICs underrepresented in health systems research.^71^
