# Supplementary material for: Coping with healthcare costs for chronic illness in low-income and middle-income countries: a systematic literature review
Source: BMJ Glob Health. 2019 Aug 21;4(4):e001475. doi: 10.1136/bmjgh-2019-001475 (PMC6730576; doi:10.1136/bmjgh-2019-001475)
Supplement: Supplementary data [file bmjgh-2019-001475supp001.pdf]

Supplementary File 1: Inclusion/Exclusion criteria

Inclusion	Exclusion
Published since 1 January 2000	**Non- LMIC country**
Includes data relating to at least one strategy for coping with out of pocket costs for dealing with chronic illness	**Does not detail a coping strategy**
Any type of study	**Non-English language**
	**Focus on general healthcare or acute illness**
