# Supplementary material for: Coping with healthcare costs for chronic illness in low-income and middle-income countries: a systematic literature review
Source: BMJ Glob Health. 2019 Aug 21;4(4):e001475. doi: 10.1136/bmjgh-2019-001475 (PMC6730576; doi:10.1136/bmjgh-2019-001475)
Supplement: Supplementary data [file bmjgh-2019-001475supp002.pdf]

Supplementary Table 2

First Author	Title	Year	Country(ies)	Study type/data source	Disease	Coping mechanisms identified	Factors influencing choice	SES differences	Evidence of financial, social or health impact
Abegunde,D O	The economic impact of chronic diseases: how do households respond to shocks? Evidence from Russia	2008	Russia	Cross-sectional survey	"Chronic disease"	Receive gifts and other transfers	i. Male head households less likely to receive transfer<br>i. Number of old people positively associated with likelihood of transfer<br>i. Chronic disease increases likelihood of transfer		
Adegoke, SA	Psychosocial burden of sickle cell disease on the family, Nigeria	2012	Nigeria	Cross-sectional survey	Sickle cell disease	i. Leave work to become caregiver<br>ii. Take out loans			

Ae-Ngibise, KA	The experience of caregivers of people living with serious mental disorders: a study from rural Ghana	2015	Ghana	Qualitative interviews and focus group discussions with caregivers	Mental disorders	i. Sell belongings (clothes, livestock)<br>ii. Take on extra work<br>iii. Depend on distant relatives for financial support			
Agatha, CO	Expenditure patterns on food and non-food items in HIV/AIDS affected and non-affected households in Kisumu District, Kenya	2010	Kenya	Cross-sectional survey	HIV/AIDS	i. Reduce food expenditure<br>ii. Sell assets			
Aji, B.	The economic impact of the insured patients with severe chronic and	2014	Indonesia	Qualitative interviews	Severe chronic or acute illness	i. Sell goods<br>ii. Borrow<br>iii. Support from family			

	acute illnesses: a qualitative approach					iv. Support from employer			
Arnold, M	Coping with the economic burden of Diabetes, TB and co-prevalence: evidence from Bishkek, Kyrgyzstan	2016	Kyrgyzstan	Cross sectional survey	Diabetes (DM)/Tuberculosis (TB)/DM and TB	i. Use income or savings<br>ii. Receive social welfare and donations<br>iii. Receive support from social networks<br>iv. Sell household assets<br>v. Borrow money	i. Significant association between employment status, age and equivalence income and choice of coping mechanism<br>ii. TB patients (younger) more likely to use income/savings<br>iii. DM patients (older, female) more	i. Non-statistically significant negative association between patients with higher wealth indices and detrimental coping strategies.	

							likely to use social welfare<br>iv. Patients who had returned from Russia due to their TB more likely to raise funds through detrimental coping strategies than patients who had not been to Russia.		
Aye, R	Factors determining household expenditure for tuberculosis and coping strategies in Tajikistan	2011	Tajikistan	Prospective longitudinal survey (4 months)	TB	i. Use credit<br>ii. Take out loan<br>iii. Sell productive asset sales (cattle, sheep, goats)			

Azzani, M	Financial burden of colorectal cancer treatment among patients and their families in a middle-income country	2016	Malaysia	Prospective longitudinal survey (1 year)	Colorectal cancer	i. Use current income and savings<br>ii. Borrow from relatives and friends (vs bank loan)<br>iii. Sell items<br>iv. Receive help (fees exemption) from Social Medical Assistance			
Bhojani, U.	Out-of-pocket healthcare payments on chronic conditions impoverish urban poor in Bangalore, India	2012	India (Bangalore)	Census and cross-sectional survey	Chronic conditions	i. Borrow money<br>ii. Sell assets<br>iii. Mortgage assets	i. Households significantly more likely to cope using savings when clinics/health centres used as	i. Households from lowest income quintile significantly more likely to borrow money than highest quintile.	

							place for consultation compared with referral hospitals and/or super-specialty hospitals)<br>ii. Households using super-specialty hospitals as place for consultation had greater odds of borrowing money than households using clinics/health centres.	ii. No households in highest income quintile had to sell and/or mortgage their assets.	

Bigdeli, M	Access to treatment for diabetes and hypertension in rural Cambodia: performance of existing social health protection schemes	2016	Cambodia	Cross-sectional survey	DM and hypertension (HTN)	i. Use income<br>ii. Borrow money<br>iii. Receive gifts from friends/family			Multiple sources of money were used when it came time to repay loans, ranging from regular income to increased labour or selling assets, livestock and land.
Cleary, S	Investigating the affordability of key health services in South Africa	2013	South Africa	Patient exit interviews	TB and HIV	i. Borrow money<br>i. Sell assets	i. Significantly higher rates of borrowing in rural areas vs urban;<br>ii. Higher percentage of users of TB and ART services borrowed or sold assets to cover costs compared		

							to obstetric care patients.		
Daivadanam, M.	Pathways to catastrophic health expenditure for acute coronary syndrome in Kerala: 'Good health at low cost'?	2012	India (Kerala)	Case studies and field notes from patient household observations	Acute Coronary Syndrome	i. Remove children from school or send to cheaper school<br>ii. Send children to work<br>iii. Send other family members to work for first time<br>iv. Move from current homes to cheaper or 'free' accommodation<br>v. Fail to comply			i. Exploitation by moneylenders was frequent, (property deeds or other assets that guarantee transactions were seized when families were unable to repay).<br>ii. Lifetime indebtedness may be passed on to the next generatio

						with treatment, choosing cheaper treatment or cutting treatment short<br>vi. Borrow from local moneylenders			n, especially in poorer households. Future prospects depleted when children drop out of school.<br>iii. Stigma of being labelled a 'charity case'.
Duraisamy, P.	Costs and financial burden of care and support services to PLHA and households in South India	2007	India (Chennai)	Cross-section of a cohort study	HIV/AIDS	i. Borrow money<br>ii. Use savings<br>iii. Sell assets<br>iv. Mortgage assets			
Foster, N	The economic burden of TB diagnosis and treatment in South Africa	2015	South Africa	Prospective cohort study (2-6 months)	TB	i. Depend on guardians or carers to take over work			

						ii. Borrow money<br>iii. Take donations<br>iv. Sell assets<br>v. Take disability grants			
Getahun, B	Tuberculosis care strategies and their economic consequences for patients: the missing link to end tuberculosis	2016	Ethiopia	Cross sectional survey	TB	i. Receive transfers from family, neighbours, friends<br>ii. Borrow money from neighbours, friends, relatives, organizations<br>iii. Sell household items			
Gustafsson-Wright, E	The inequitable impact of health shocks on the uninsured in Namibia	2011	Namibia	Cross-sectional survey	HIV/AIDS	i. Take grants, gifts<br>ii. Sell assets<br>iii. Decrease consumption	Households with private insurance less likely to engage in these coping strategies.		

						iv. Take loans			
Huffman, M	A cross-sectional study of the microeconomic impact of cardiovascular disease hospitalization in four low- and middle-income countries	2011	Argentina, China, India, Tanzania	Cross-sectional survey	CVD	Distress financing (borrowing money from relatives/friends, taking loans from banks/other lenders, or selling assets (e.g.property)		Poor more likely to engage in distress financing.	
Hutchinson, C	Financial barriers and coping strategies: a qualitative study of accessing multidrug-resistant tuberculosis and tuberculosis care in Yunnan, China	2017	China	Qualitative interviews	TB and MDR-TB	i. Use savings<br>ii. Sell assets (livestock, crops)<br>iii. Borrow from financial institution<br>iv. Receive informal transfers, gifts or loans			Exhaustion of resources<br>Reduced consumption<br>Compromises in medication<br>Strained relationships<br>Stigma, shame<br>(Can't say these are long term based on longitudinal

						from family<br>v. Use replacement labour (e.g. send another family member to work in place of ill member)<br>vi. Take a donation from healthcare provider			data because study is qual)
Jackson, S	Poverty and the economic effects of TB in rural China	2006	China	Cross-sectional survey (TB cases and sex-matched controls)	TB	i. Borrow from relatives and friends<br>ii. Borrow from banks<br>ii. Sell productive assets (e.g. tractors,			Poverty a factor in discontinuing treatment.

						draft animals.)			
Karan, A	The household-level economic burden of heart disease in India	2015	India	Cross-sectional survey (CVD households matched to non-CVD households)	CVD	i. Borrow money<br>ii. Sell assets	CVD patients more likely to borrow and sell assets to finance care than non-CVD-affected households.		
Kastor, A	Disease-specific out-of-pocket and catastrophic health expenditure on hospitalization in India: Do Indian households face distress health financing?	2018	India	Cross-sectional survey	NCDs	Distress financing, defined as borrowing money, sale of property/ assets or contributions from friends and relatives	i. Cancer, TB, heart disease and injuries showed higher odds of distress financing compared to communicable disease<br>ii. Odds of distress	Odds of distress financing declined with increased wealth	

							financing declined with higher educational level<br>iii. Likelihood of distress financing higher among inpatients admitted in private hospitals vs public hospitals		
Kavosi, Z	Catastrophic health expenditures and coping strategies in households with cancer patients in Shiraz Namazi Hospital	2014	Iran	Cross-sectional survey	Cancer	i. Use current income<br>ii. Receive insurance reimbursement<br>iii. Use savings<br>iv. Borrow money<br>v. Sell properties	Use of income, savings and insurance more likely among those who do not experience catastrophic spending.		

Laokri, A	Assessing the economic burden of illness for tuberculosis patients in Benin: determinants and consequences of catastrophic health expenditures and inequities	2014	Benin	Cross-sectional survey	TB	i. Deplete savings<br>ii. Borrow money<br>iii. Sell assets (e.g. cattle, seeds, piece of land)<br>iv. Cut budgets (e.g. housing, education)	Higher-income households more likely to deplete savings while lower-income households more likely to become indebted.		
Laokri, A	Catastrophic out-of-pocket health costs due to inefficient management of the health-care system: an evidence-based case study of tuberculosis control program in rural Burkina Faso	2011	Burkina Faso	Cross-sectional survey	TB	i. Incur debts<br>ii. Sell cattle, seeds for following year's sowing or land<br>iii. Take loans from family or community members			In some cases households depleted all their savings on TB treatment

Lonnroth, K	Social franchising of TB care through private GPs in Myanmar: an assessment of treatment results, access, equity and financial protection	2007	Myanmar	Prospective (facility-based) longitudinal survey (6 months)	TB	Borrow money	Higher proportion of poor had to borrow money compared with higher income patients.		
Lopera, MM	Out-of-pocket expenditures and coping strategies for people living with HIV: Bogota, Colombia, 2009	2011	Colombia	Cross-sectional survey	HIV	i. Ask extended family or friends to take care of children permanently<br>ii. Borrow from friends<br>iii. Default on debts<br>iv. Sell assets or properties<br>v. Reduce food consumption			

						vi. Reduce quality of life<br>vii. Institutionalize HIV patient<br>viii. Remove children from school<br>ix. Borrow from institutions<br>x. Take on more work to cover expenses<br>xi. Institutionalize children without HIV<br>xii. Stop working to take care of person with HIV (vs. paying for a			

						caregiver)			
Maneeta, J	Economic burden of breast cancer to the households in Punjab, India	2016	India (Punjab)	Cross-sectional survey	Breast cancer	i. Borrow money<br>ii. Receive support from social network<br>iii. Use savings<br>iv. Sell financial assets<br>v. Delay payment of pre-existing loans<br>vi. Sell economically productive assets<br>vii. Rent out rooms in house<br>viii. Delay payment of bills<br>ix. Pawn jewellery<br>x. Borrow at high	i. Patients with stage 2 cancer or above more likely to engage in distress financing compared to earlier stages<br>ii. Urban households less likely to face distress financing compared to rural households.	i. Households with lower per capita income more likely to use 'distress financing' than higher income households.<br>ii. Lower annual household income significantly associated with reduced food expenditure, reduced expenditure on education, reduced	

						rate of interest<br>xi. Take credit from a local shop<br>xii. Take financial aid (from govt or NGO)<br>xiii. Early entry into labour market		expenditure on social events, and early entry into labour market.<br>iii. None of these strategies were adopted by upper income households.	
Mauch, V	Assessing access barriers to tuberculosis care with the tool to Estimate Patients' Costs: pilot results from two districts in Kenya	2011	Kenya	Cross-sectional survey (facility based)	TB	i. Borrow money<br>ii. Sell assets (mostly livestock)		Middle-income patients selling significantly more than low- or high-income patients. (Those who sold assets gained significantly less than the market price estimated by the patient).	

Mauch, V	Free tuberculosis diagnosis and treatment are not enough: patient cost evidence from three continents	2013	Ghana, Vietnam, Dominican Republic	Cross-sectional survey	TB	i. Sell property (land, livestock, other)<br>ii. Take out loans (at interest >10% or without interest)	Differences by country: selling property most common in Ghana; borrowing money most common in Ghana and Vietnam.		
Muniyandi, M	Costs to patients with tuberculosis treated under DOTS programme.	2005	India	Cross-sectional survey (facility based)	TB	Borrow money			

Murphy, A	The economic burden of chronic disease care faced by households in Ukraine: a cross-sectional matching study of angina patients	2013	Ukraine	Cross-sectional survey (angina patients matched to controls)	Angina	i. Use income<br>ii. Use savings<br>iii. Sell assets<br>iv. Borrow from friends or family<br>v. Borrow from others	Odds of engaging in 'sale of assets' to finance OPP significantly higher among angina households (vs. households without angina)		
Mutyambizi, V	An exploratory study of the resources used by, and the coping strategies of poor urban households affected by HIV/AIDS in Harare City	2002	Zimbabwe	Cross-sectional survey and focus group discussions and	HIV/AIDS	i. Borrow money<br>ii. Reduce expenditure<br>iii. Seek alternative care<br>iv. Sell assets<br>v. Receive NGO support<br>vi. Seek cheaper provider<br>vii. Stop treatment<br>viii. Delay treatment	Strategy differed according to social capital available (e.g. NGO support)	Lower income community had higher social capital and used social network resources more often.	

						ix. Receive social welfare or medical aid<br>x. Use savings			
Oni, SA	The economic impact of HIV/AIDS on rural households in Limpopo province	2002	South Africa	Cross-sectional survey	HIV/AIDS	i. Sell household assets<br>ii. Withdraw children from school<br>iii. Join community support groups			
Oluwagbemiga, AE	HIV/AIDS and family support systems: a situation analysis of people living with HIV/AIDS in Lagos State	2007	Nigeria	Focus group discussions and in-depth interviews	HIV/AIDS	i. Sell property<br>ii. Take children out of school			

Palmer, M	The Economic Lives of People with Disabilities in Vietnam	2015	Vietnam	Focus group discussions	Disability	i. Borrow (from money lenders, local organizations, family and friends)<br>ii. Draw on savings<br>iii. Substitute labour within household<br>iv. Cut-back on, or forego, disability-related expenditures			
Palmer, M	Healthcare utilization, cost burden and coping strategies by disability status: an analysis of the Viet Nam National	2011	Vietnam	Cross-sectional survey	Disability	i. Use income<br>ii. Use savings<br>iii. Borrow money<br>iv. Sell home produce<br>v. Work more to			

	Health Survey					increase income<br>vi. Receive financial support from outside household			
Perera, M	Falling into the medical poverty trap in Sri Lanka: what can be done?	2007	Sri Lanka	Focus group discussions and in-depth interviews	DM	i. Pawn or sell assets (often income earning assets such as land or businesses)<br>ii. Take out loans<br>iii. Take children out of school<br>iv. Receive financial or in-kind assistance from government poverty alleviation			i. Income-earning potential diminished due to sale of income-earning assets.<br>ii. Long-term nature of diabetes costs pushed households into poverty and prevented recovery.<br>iii. Healthcare cost burden exacerbated poor

						programs, employers, family members or community			health - when households could no longer afford treatment, likelihood of recurrent acute phases increased, in turn leading to higher healthcare costs.
Prakongsai, P	The implications of benefit package design: the impact on poor Thai households of excluding renal replacement therapy. (Special Issue: Managing risks:	2009	Thailand	Case studies (semi-structured and in-depth interviews, and direct observation).	Renal replacement therapy for End-stage renal disease.	i. Reduce frequency of dialyses<br>ii. Reduce food consumption<br>iii. Use public transportation to hospitals<br>iv. Take high		Strategies more common among poor.	

	household illness costs, coping strategies and access to social protection.)					interest loans			
Prasanna, T	Catastrophic costs of tuberculosis care: a mixed methods study from Puducherry, India	2018	India (Puducherry)	Cross-sectional survey and qualitative interviews with patients	TB	i. Borrow money<br>ii. Sell household property<br>iii. Pledge jewels<br>iv. Dissolve savings			
Ramma, L	Patients' costs associated with seeking and accessing treatment for drug-resistant	2015	South Africa	Cross-sectional survey	Drug resistant TB	Loans			

	tuberculosis in South Africa								
Saqib, SE	Economic burden of tuberculosis and its coping mechanismat the household level in Pakistan	2018	Pakistan	Cross-sectional survey	TB	i. Borrow money<br>ii. Sell productive assets (e.g. cattle, gold,silver,property)<br>iii. Withdraw children from school<br>iv. Reduce household expenditure on daily needs<br>v. Use household savings<br>vi. Take on additional		i. Borrowing money, selling assets, withdrawing children from school and taking on extra work more common among lower-income households<br>ii. Use of savings more common among higher-income households.	

						working hours, including overtime work and second jobs<br>vii. Receive religious aid			
Schneider, P	Paying for HIV/AIDS services—lessons from National Health Accounts and community-based health insurance in Rwanda, 1998–1999.	2001	Rwanda	Cross-sectional survey	HIV/AIDS	i. Receive assistance (from family/friend, church, employer, health insurance, international organization, NGO, other private sources)<br>ii. Borrow money<br>iii. Sell assets			
Sukeri, S	Does tax-based health financing offer	2017	Malaysia	Cross-sectional survey	Ischemic heart disease	i. Borrow money<br>ii. Sell assets			

	protection from financial catastrophe? Findings from a household economic impact survey of ischaemic heart disease in Malaysia								
Tolla, MT	Out-of-pocket expenditures for prevention and treatment of cardiovascular disease in general and specialised cardiac hospitals in Addis Ababa, Ethiopia: a cross-sectional cohort study	2017	Ethiopia	Cross-sectional survey	Ischemic heart disease, stroke, hypertension, dyslipidemia	i. Use savings<br>ii. Receive family support<br>iii. Borrow money<br>iv. Sell assets	Coping strategies more common for inpatient care compared with outpatient care.		

Uddin, MJ	Consequences of hypertension and chronic obstructive pulmonary disease, healthcare-seeking behaviors of patients, and responses of the health system: a population-based cross-sectional study in Bangladesh	2014	Bangladesh	Cross-sectional survey and qualitative interviews	HTN and Chronic Obstructive Pulmonary Disease	i. Borrow from friend or relative<br>ii. Reduce expenditure on food or other items<br>iii. Use savings<br>iv. Sell assets			
Ukwaja, K. N.	The high cost of free tuberculosis services: patient and household costs associated with tuberculosis care in Ebonyi State, Nigeria	2013	Nigeria	Cross-sectional survey	TB	i. Borrow money<br>ii. Sell assets<br>iii. Borrow money and sell assets			

Wafula, CO	Economic impact of HIV/AIDS on rural households in Suba Distict, Kenya	2013	Kenya	Cross-sectional survey	HIV/AIDS	i. Withdraw savings<br>ii. Sell assets (cattle)	i. Likelihood of withdrawing savings highest among households with patients at more advanced stages of disease.<br>ii. Cattle sale increased with advanced stages of HIV.		
